# Estimated radiation risk of cancer from dental cone-beam computed tomography imaging in orthodontics patients

**DOI:** 10.1186/s12903-018-0592-5

**Published:** 2018-08-03

**Authors:** Jih-Kuei Yeh, Chia-Hui Chen

**Affiliations:** 1 Division of Family Medicine, Kaohsiung Municipal United Hospital, No.976, Jhonghua 1st Rd., Gushan Dist, Kaohsiung City, 804 Taiwan; 2 Department of Medical Imaging and Radiological Science, Central Taiwan University of Science and Technology, No.666, Buzih Road, Beitun District, Taichung City, 40601 Taiwan; 3 College of Photonics National Chiao Tung University, No.301, Gaofa 3rd Rd., Guiren Dist, Tainan City, 711 Taiwan

**Keywords:** Cone-beam computed tomography (CBCT), Organ doses, Monte Carlo simulation, PCXMC, Risk of exposure-induced death (REID)

## Abstract

**Background:**

Radiation dose evaluation is important to cone-beam computed tomography (CBCT) for routine orthodontic treatment planning, especially for a significant proportion of children in orthodontic patients. This study evaluated the patient radiation dose and estimated the radiation cancer risk on dental CBCT according to the calculations by the Monte Carlo simulation method.

**Methods:**

The dental CBCT scanner evaluated in this project was the i- CAT® (Imaging Sciences International Inc., PA, U.S.A.) device. Organ doses and effective doses were calculated by using personal computer-based Monte Carlo simulation (PCXMC 2.0 Rotation) software. The cancer risk resulting from the exposure to ionizing radiation was estimated by using the BEIR VII (Biologic Effects of Ionizing Radiation VII) report model, and the risk of exposure-induced death (REID) was assessed by PCXMC 2.0 Rotation software.

**Results:**

The largest contribution to the organ dose and effective dose at Zref 83 cm positioned in the dental CBCT x-ray beam centerline was from the salivary glands (738.29μGy, 7.38 μSv). The different organ doses showed the maximum values at the different Zref locations, and the largest contribution to the organ dose and effective dose of all simulated positions was from the thyroid (928.77μGy, 37.5 μSv). The REID values in the 10-year olds (22.6 × 10^− 7^, female; 19 × 10^− 7^, male) were approximately double than those in 30-year olds (10.4 × 10^− 7^, female; 8.88 × 10^− 7^, male) for all cancers. The highest change during age range from 10 to 30 was shown in breast cancer of females.

**Conclusions:**

Although individual cancer risk estimates as a function of gender and age are small, the concern about the risks from dental CBCT is related to the rapid increase in its use for orthodontic practice, especially in children patients.

## Background

In recent years, a vast amount of dental cone-beam computed tomography (CBCT) devices has become available and is now a commonly used imaging modality for clinical indications in dentistry [1, 2]. As compared with traditional radiographs, CBCT supporting an overview of three-dimensional imaging is a relatively new imaging technology with proven usefulness in imaging of hard tissues in dentistry. More accurate diagnosis of skeletal asymmetry, easier location of impacted teeth, improved surgical planning, and increased detection of pathologies by using dental CBCT have all been reported [3, 4]. Therefore, dental CBCT has more frequently become an alternative imaging modality for many orthodontic clinics.

Questions about the amount of patient exposure dose in diagnostic dental CBCT examination remain an important issue. Roberts JA et al. have indicated that CBCT delivers a higher dose to the patient than a typical panoramic radiograph by 5–16 times [5]. Several studies comparing the radiation dose of CBCT to those of other dental modalities have concluded that conventional images still deliver the lowest doses to patients [6–9] . Al Najjar A et al. have reported that CBCT exposure settings for children and adults have significantly higher equivalent radiation doses to the head and neck organs in children than in adults [10] . Radiation dose evaluation is important to CBCT for routine orthodontic treatment planning, especially for a significant proportion of children as orthodontic patients.

There are several techniques to evaluate the effective dose quantity by using dosimeters inserted in anthropomorphic phantoms, such as thermoluminescent dosimeter (TLD) [11, 12] and optically stimulated luminescent dosimeter [10, 13] . Also, the use of radiochromic film [14] and metal-oxide semiconductor field-effect transistor dosimeter [15] offers a very fast readout possibility compared with very time-consuming dosimeter; however, these methods require several steps for data acquisition and also have significant associated uncertainties [16, 17] .

Another option is the Monte Carlo method of computer simulation to quantify the exposure conditions for numerous radiological imaging techniques [18, 19] . The personal computer-based Monte Carlo (PCXMC) software is a Monte Carlo simulation application adapted for use in the personal computer developed by the Radiation and Nuclear Safety Authority in Finland [20]. The supplemental program (PCXMC 2.0 Ration) for rotational technique allows dose calculations in cases where the x-ray system has a center point of rotation, and the radiation is aimed to the patient from various directions so that the central axis of the beam goes through the reference point of PCXMC [21]. Great concern about the stochastic effects of radiation including carcinogenesis and genetic mutations is considered. The probability of an effect on cancer risk is proportional to the exposure radiation dose. The patient doses in dental CBCT examinations can be calculated by using PCXMX 2.0 Rotation software; another approach for the estimation of cancer risk.

The purpose of this study was to evaluate the patient radiation dose and estimate the radiation cancer risk on dental CBCT according to the calculations by the computer simulation with PCXMC 2.0 Rotation software.

## Methods

### CBCT scanner

The CBCT scanner evaluated in this project was the i- CAT® (Imaging Sciences International Inc., PA, U.S.A.). This CBCT device constructs a three-dimensional model from images taken during a rotational X-Ray sequence. It has several protocols defined by the manufacturer. The protocol was used by tube voltage 120 kV, 18.54 mAs, 16× 13 cm field of view (FOV), and voxel size 0.4 mm in this study.

### PCXMC simulation

**Fig. 1:**
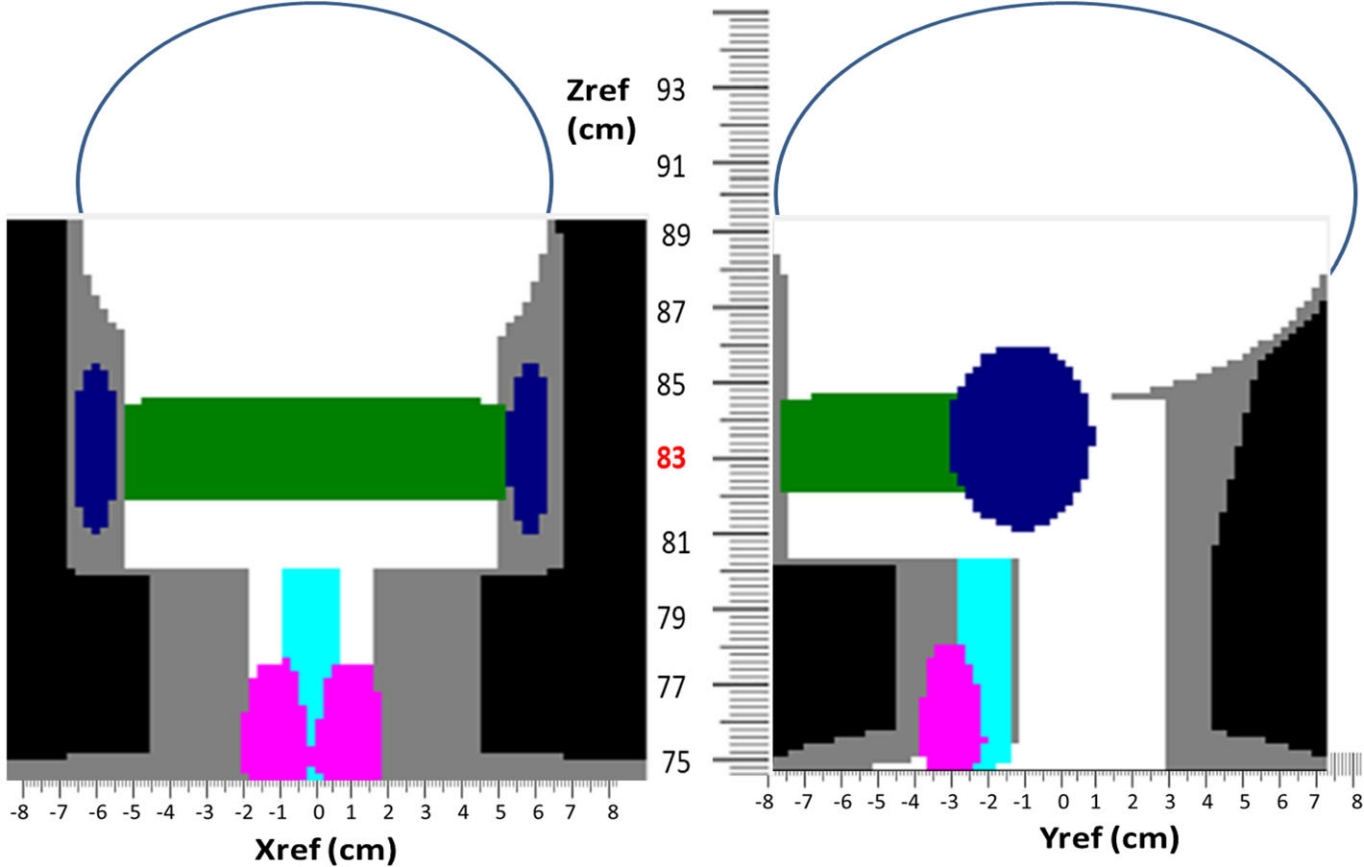
An example of geometry for generating the rotation and consequent calculation data. Zref 83.0 cm (red) for the CBCT x-ray beam centerline

In this study, PCXMC 2.0 Ration (STUK, Helsinki, Finland) was used for calculating the organ doses and effective doses of simulation phantom in medical x-ray examinations and no patient was directly involved. The program incorporates adjustable-size pediatric and adult phantom models and allows a free choice of the x-ray examination technique. The doses were calculated in 29 organs and tissues specified by the International Commission on Radiological Protection (ICRP) dosimetry recommendations, and the effective doses with the tissue weighting factors based on ICRP publication 103 [22]. In PCXMC 2.0 Rotation program, ‘Xref’, ‘Yref’, and ‘Zref’ are the coordinates of a point inside the phantom, through which the central axis of the x-ray beam is directed so that positive z axis points upwards, the x axis is to the left-hand side, and the y axis is to the back of the phantom (Fig. 1). The necessary input data for simulation of execution are patient data (simulation age, gender, height, and mass), beam parameters, and irradiation geometry. The parameters for the software PCXMC 2.0 Rotation include 360 degree rotation, 120 kV, 14 mm AL filter, X-ray beam width16 cm, X-ray beam height 8 cm, Xref 0 cm, Yref − 5 cm, and Zref variable. These radiosensitive organs, including brain, esophagus, salivary glands, and thyroid, from neck area to head correspond roughly to Zref values of 75.0–92.5 cm on the PCXMC 2.0 Rotation simulations. The reference point Zref of 83.0 cm is positioned in the CBCT x-ray beam centerline. The rotation axis of the simulations is set to Yref of − 5 cm to cover the oral cavity volume. The focus distance reference (FRD) setting 52 cm is the distance from the focal point to the center of the FOV.

### Estimates of radiation risk

The cancer risk from the exposure to ionizing radiation was estimated by using the BEIR VII (Biologic Effects of Ionizing Radiation VII) report model [23] and the PCXMC 2.0 Ration software. An important task of the BEIR VII committee is to develop the risk models for estimating the cancer risk for an exposed individual. This model applies the linear no-threshold (LNT) model, stating that for low levels of low linear energy transfer ionizing radiation, a minor increase in cancer risk can potentially cause an individual to develop cancer. The task requires expressing the dependence of risk not only on radiation dose, but on sex and age at exposure as well. The BEIR VII committee has derived risk models both for cancer incidence and for cancer mortality. Age-dependent mortality rates are used for subsequent assessment of lifetime cancer risk. For all cancer types, the BEIR VII committee derived absolute and relative risk models. In the absolute risk model, excess cancer risk from radiation is independent of the background cancer risk. Otherwise, the radiation risk is proportional to the background cancer risk in the relative risk model. The excess risk values are the basis of the lifetime risk estimates. In PCXMC 2.0 Rotation software, lifetime risks are expressed in terms of risk of exposure-induced death (REID) as assessed by the data including age, gender and mortality statistics (Asian) of the patient in the program. According to the BEIR VII risk model, cancer induction as a result of exposure to radiation is thought by most to occur in a stochastic manner. There is no threshold point and risk increases in a linear-quadratic fashion with dose. The REID value depends on the BEIR VII risk model. The risk models for leukemia, colon cancer, liver cancer, lung cancer, stomach cancer, breast cancer and other cancers were analyzed in our study. Except for breast cancer, the REID values of the individual cancers were reported for both genders. The t-test for data analysis was performed by SPSS 19.0 for Windows (SPSS, Chicago, IL).

## Results

**Fig. 2:**
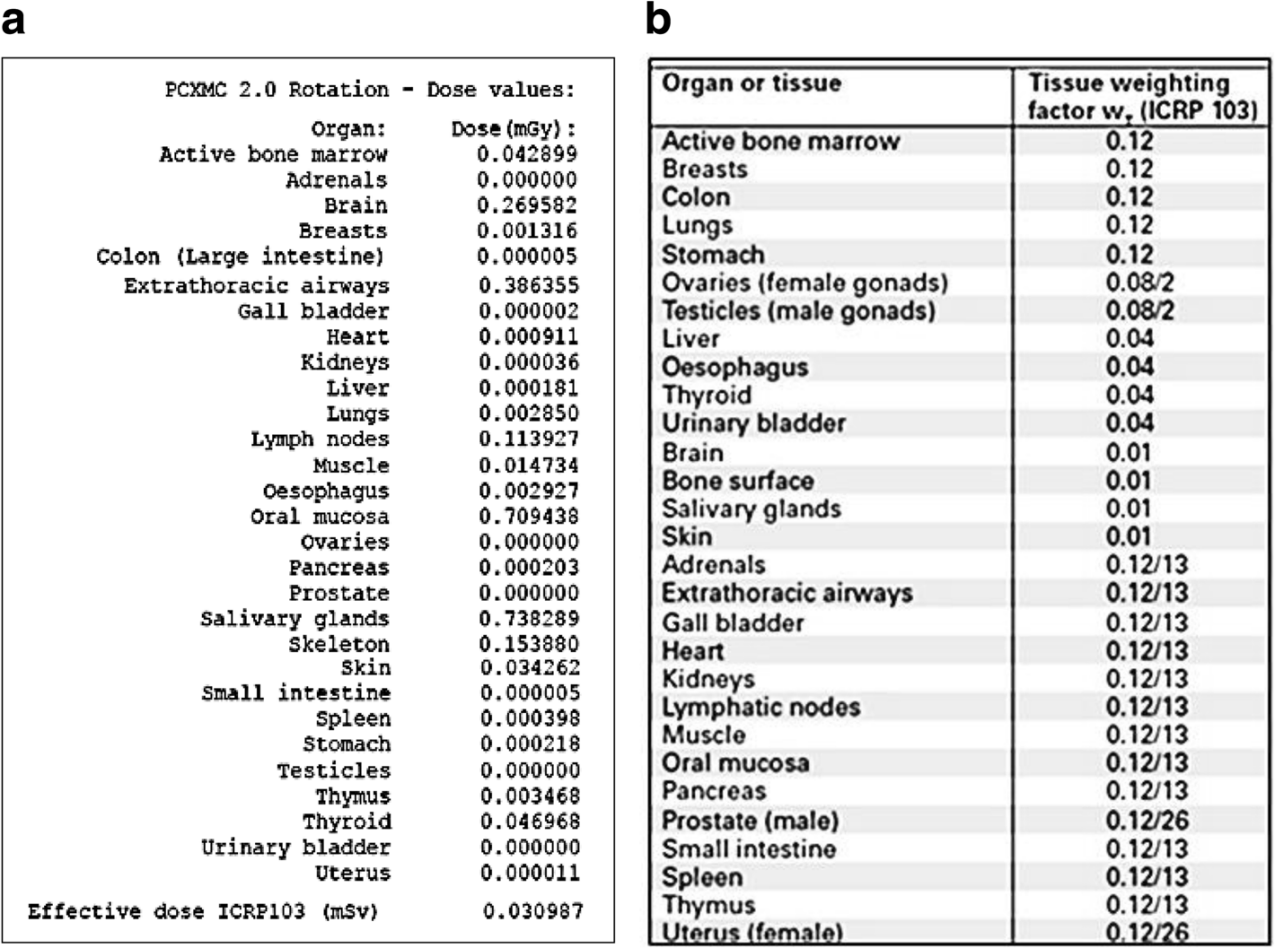
PCXMC 2.0 Rotation reporting a) all 29 organ doses and total effective dose according to ICRP 103 at centerline, and b) different tissue weighting factors for the calculation of the effective dose according to ICRP 103

**Fig. 3:**
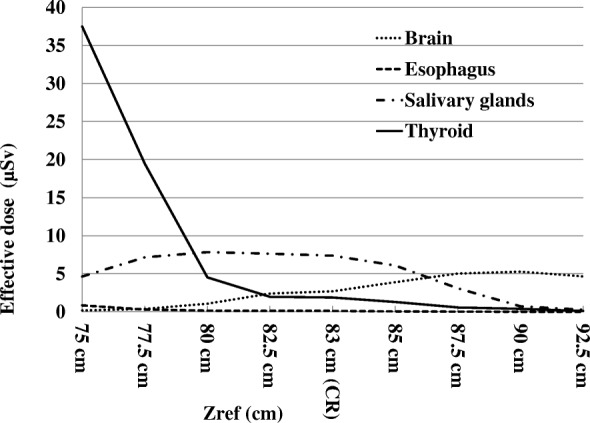
Effective dose simulations ranging from the neck to head (Zref 75.0–92.5 cm)

Fig. 2 shows the all 29 organ doses and total effective dose according to ICRP 103 by the Monte Carlo PCXMC 2.0 Ration software simulation at the centerline with the reference point Zref at 83.0 cm. The organs for the radiation dose on dental CBCT included brain, esophagus, salivary glands, and thyroid. The results of these organs with the reference point Zref at 83.0 cm, and in the measured height range from the neck to head with the Zref coordinating from 75.0 to 92.5 cm were presented in Table 1. The largest contribution to the organ dose was from the salivary glands (738.29μGy) and brain organ dosage was the second largest (269.58μGy) at Zref 83.0 cm. The overall organ dose variation range was from 928.77μGy (thyroid) at Zref 75 cm to 0.5μGy (esophagus) at Zref 92.5 cm. Fig. 3 and Table 2 show the effective dose variations by the PCXMC software simulation in the measured height range from the neck to head with the Zref coordinating from 75.0 to 92.5 cm, and at Zref 83.0 cm. The highest contribution to the effective dose of all simulated positions was from the thyroid (37.50 μSv) at Zref 75.0 cm. The brain had the highest contribution to the effective dose of 5.27 μSv at Zref 90.0 cm, esophagus effective dose 0.85 μSv at Zref 75.0 cm, and salivary glands 7.84 μSv at Zref 80.0 cm. The total effective dose was 30.99 μSv in the dental CBCT x-ray beam centerline.

**Table 1:** Monte Carlo PCXMC estimates of organ dose on the variation of Zref

	Organ Dose (μGy)								
Organ/Zref.	75 cm	77.5 cm	80 cm	82.5 cm	83 cm (CL)	85 cm	87.5 cm	90 cm	92.5 cm
Brain	18.37	38.27	107.35	239.67	269.58	389.1	501.96	527.39	465.4
Esophagus	21.2	7.53	3.86	2.79	2.93	1.55	0.47	0.36	0.5
Salivary glands	463.58	715.93	783.88	763.54	738.29	609.18	308.01	71.91	26.6
Thyroid	928.77	486.49	113.07	49.54	46.97	32.25	14.39	9.65	2.46

*CL* centerline

**Table 2:** Effective Dose (μSv) according to ICRP 103 on the variation of Zref

Organ/Zref.	ICRP 103 W_T_	Effective Dose (μSv)								
		75 cm	77.5 cm	80 cm	82.5 cm	83 cm (CL)	85 cm	87.5 cm	90 cm	92.5 cm
Brain	0.01	0.18	0.38	1.07	2.40	2.70	3.89	5.02	5.27	4.65
Esophagus	0.04	0.85	0.30	0.15	0.11	0.12	0.06	0.02	0.01	0.01
Salivary glands	0.01	4.64	7.16	7.84	7.64	7.38	6.09	3.08	0.72	0.27
Thyroid	0.04	37.50	19.46	4.52	1.98	1.88	1.29	0.58	0.39	0.10

*CL* centerline

**Fig. 4:**
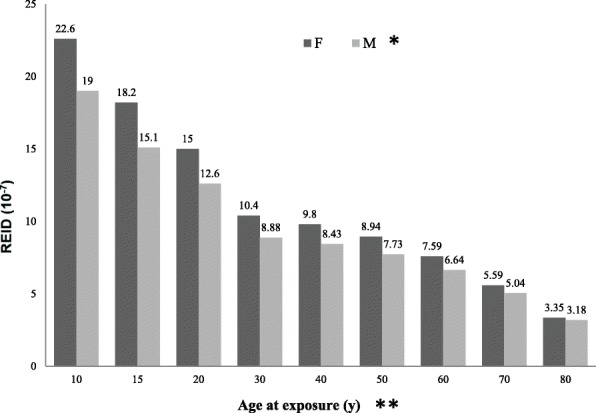
REID (10^− 7^) based on dental CBCT scans for female and male patients as a function of age. *significant difference in gender (*P* = 0.03), **significant difference in different ages of exposure (*P* = 0.01, female; *P* < 0.0001, male)

**Fig. 5:**
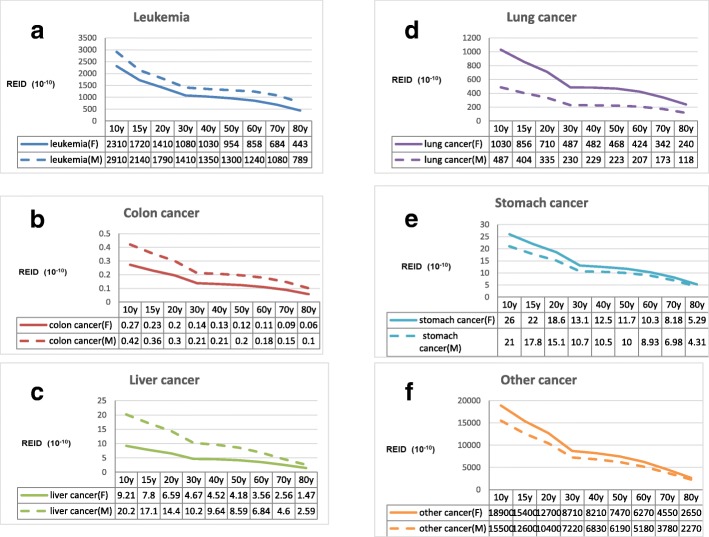
The REID values for the six individual radiogenic cancers, including **a** leukemia, **b** colon cancer, **c** liver cancer, **d** lung cancer, **e** stomach cancer, and (**f**) other cancer, for male and female patients as a function of age

**Fig. 6:**
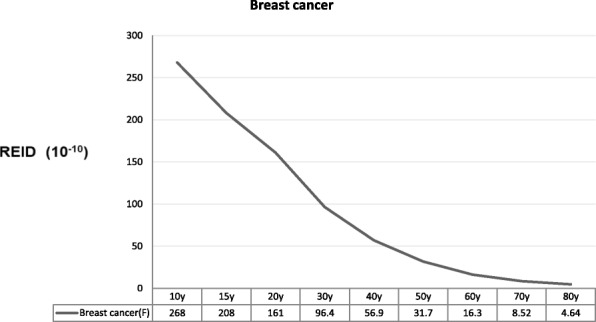
The REID values for breast cancer for female patients as a function of age

The REID results of dental CBCT scan as a function of age for male and female patients are shown in Fig. 4. The REID values were considerably higher in females than males (*P* = 0.03), and the radiation risk decreased with increasing age in both genders (*P* = 0.01, female; *P* < 0.0001, male). The decrease of radiation risk became smoother after the 30-year-old subjects. The highest REID values were in the 10-year-old subjects with 22.6 × 10^− 7^ in females and 19 × 10^− 7^ in males. The six individual radiogenic cancers, including leukemia, colon cancer, liver cancer, lung cancer, stomach cancer, and other cancer, were analyzed for both genders (Fig. 5 a-f). The REID values for the six individual cancers did not monotonically decrease with increasing age at exposure with the gradually small change after 30 years of age. The radiation-induced cancer risks for leukemia, colon cancer, and liver cancer were higher in males than females; however, the risks for lung cancer, stomach cancer, and other cancer were higher in females than males. The radiation-induced breast cancer risks decreased monotonically with increasing age at exposure (Fig. 6). The REID from breast cancer was highest at the 10-year-old level.

## Discussion

In this study, we used the PCXMC 2.0 Rotation software to calculate the patient radiation dose and estimate cancer risk on dental CBCT. Our findings demonstrated that the largest contribution to the organ dose and effective dose was from the salivary glands at Zref 83 cm positioned in the CBCT x-ray beam centerline. The different organ doses showed the maximum values at the different Zref locations; the largest contribution to the organ dose and effective dose of all simulated positions was from the thyroid. We observed the decreased REID values with increasing age at exposure for all cancers in both genders.

When three-dimensional imaging is required in orthodontic practice, dental CBCT has developed to replace the traditional panoramic and lateral cephalometric radiographs taken for orthodontic diagnosis and treatment planning. From a radiation-protection point of view, the effective dose is lower for the conventional radiographs than for CT. The tissues in the head and neck regions have a wide range of weighted factors to radiation exposure; therefore, the increase in exposure of more weighted tissues will result in disproportionate increase in effective dosage. Moreover, the increased usage of CBCT technology in dental and maxillofacial radiology has led to concern about radiation exposure. Although measurement of the radiation exposure by TLD is the most popular method concerning effective dosage, a major drawback is the need to replace the TLDs after every exposure. The high variations in the effective doses of all radiosensitive organs by TLD between these studies can be caused by the use of different phantoms, and different numbers of dosimeters, and different locations of dosimeters [11, 12, 24]. The Monte Carlo method is another way to measure the effective dosage. The simulation of the pathway of X-ray photons as they interact with organ tissue can be computed in comparison with the dosimeters. Our results from the Monte Carlo method on dental CBCT showed that both maximum organ dose and effective dose were from the salivary glands in the x-ray beam centerline. In the ICRP 103 2007 publication with new factors and explicitness, the salivary glands should be specially considered in dentistry radiology.

In an early study with the different CBCT scanner performed by Koivisto J et al. [15], the highest contribution to the effective dose was from the thyroid gland at Zref 74.0 cm, and the salivary glands had the highest contribution to the effective dose at Zref 83.0 cm. The effective doses are strongly dependent on the chosen beam centerline height positions. In our study, we observed similar results with lower effective doses. The major differences between the results attained by Koivisto J et al. and our study were due to the larger FOV, higher kV, and lower mAs in our dental CBCT examination. Several studies have suggested that the technique with a significant reduction in exposure mAs can yield a corresponding reduction in dose and in risk [25]. Nevertheless, the curves of simulated effective dose as a function of Zref were similar to the results attained in their study.

In regard to radiation exposure, the REID values in the 10-year-old subjects were approximately double those in the 30-year-old subjects for all cancers. The highest change was shown in breast cancer of females. Cancer risks decrease with increasing age because children have more years of life during which a potential cancer can be expressed. Since the growing children have a larger proportion of dividing cells, they are inherently more vulnerable to radiation. Moreover, the smaller body size of children infers a further potential increase in risk than that of adults because the adjacent organs receive larger doses of the scatter radiation. The radiation sensitivity of the breast is increased in girls aged 10 to 20 years during which breast tissue is undergoing rapid cell proliferation [26]. In cancer risk assessment, the results of our study were not inconsistency with those of the other related studies. Pauwels et al. have estimated cancer risk from CBCT exposures by using the 2 different scanners, SCANORA 3D (Soredex, Tuusula, Finland) and NewTom 9000 (QR, Verona, Italy) [27]. They use 8 TLDs attaching to the patient’s skin at the standardized locations, then convert skin doses to organ doses by the correlation factors. They have concluded that the probability to develop a radiation-induced cancer vary between 2.7 × 10^− 6^ (age > 60) and 9.8 × 10^− 6^ (age 8–11) with an average of 6.0 × 10^− 6^. In our study, REID values were calculated from the radiosensitive organ doses by using the PCXMC 2.0 Rotation software. The discrepancy between the 2 studies can be possible because of the different methods.

Some challenge argues that the BEIR risk estimates of medical imaging are derived from the organ doses involved and organ-specific cancer incidence or mortality data of atomic-bomb survivors in Japan. Epidemiologic data of the BERI risk model are limited, and greatly different from the population of individuals for dental CBCT imaging. For the purpose of risk estimation, doses to patients have been converted to effective doses. The ICRP has warned against the use of effective dose for epidemiologic studies or for estimation of individual risks [28]. Radiation hormesis is the hypothesis that low doses of radiation are beneficial or radiation-activated natural protection [29, 30]. The effects of low-dose ionizing radiation from the medical imaging, like dental CBCT, are difficult to observe. Reports by the United Nations Scientific Committee on the Effects of Atomic Radiation argue that there is no evidence for hormesis in humans [31]. Based on the LNT risk assessment model, any amount of radiation exposure may lead to cancer in a population by the stochastic biological effects from ionizing radiation. Calabrese, EJ et al. have explored the origin of the LNT dose-response model and the utility of the model in cancer risk assessment worldwide [38]. Therefore, the LNT dose-response model is generally used to estimate cancer risks from exposures to low-level radiation. In addition, some limited evidence has shown the increase of radiation-related tumor in the brain and thyroid glands [32]. Indeed, we suggest that routinely wearing a lead apron can protect these organs away from the primary beam or even from the scattered radiation of CBCT. Another limitation of our study was that the relationship between radiation dose and FOV was not included. The different FOV settings by different CBCT units can provide different anatomic coverage of the radiosentive organs in head and neck regions.

## Conclusions

Findings from our study showed that the REID values decreased with increasing age at exposure for all cancers. Although individual cancer risk estimates as a function of gender and age are small, the concern about the risks from dental CBCT is related to the rapid increase in its use for orthodontic practice, especially in children patients.

## Abbreviations

BEIR VII: Biologic Effects of Ionizing Radiation VII
CBCT: cone-beam computed tomography
FOV: field of view
FRD: focus distance reference
ICRP: International Commission on Radiological Protection
REID: risk of exposure-induced death
TLD: thermoluminescent dosimeter
